# Role of Non-Coding RNAs in Lung Circadian Clock Related Diseases

**DOI:** 10.3390/ijms21083013

**Published:** 2020-04-24

**Authors:** Srinivasan Chinnapaiyan, Rajib Kumar Dutta, Dinesh Devadoss, Hitendra S Chand, Irfan Rahman, Hoshang Jehangir Unwalla

**Affiliations:** 1 Department of Immunology and Nano-Medicine, Herbert Wertheim College of Medicine, Florida International University, Miami, FL 33199, USA; schinnap@fiu.edu (S.C.); ddevados@fiu.edu (D.D.); hchand@fiu.edi (H.S.C.); 2 School of Medicine and Dentistry, University of Rochester Medical Center, Rochester, NY 14642, USA; Irfan_Rahman@urmc.rochester.edu

**Keywords:** lung circadian clock, non-coding RNA, COPD, asthma, cigarette smoke

## Abstract

Circadian oscillations are regulated at both central and peripheral levels to maintain physiological homeostasis. The central circadian clock consists of a central pacemaker in the suprachiasmatic nucleus that is entrained by light dark cycles and this, in turn, synchronizes the peripheral clock inherent in other organs. Circadian dysregulation has been attributed to dysregulation of peripheral clock and also associated with several diseases. Components of the molecular clock are disrupted in lung diseases like chronic obstructive pulmonary disease (COPD), asthma and IPF. Airway epithelial cells play an important role in temporally organizing magnitude of immune response, DNA damage response and acute airway inflammation. Non-coding RNAs play an important role in regulation of molecular clock and in turn are also regulated by clock components. Dysregulation of these non-coding RNAs have been shown to impact the expression of core clock genes as well as clock output genes in many organs. However, no studies have currently looked at the potential impact of these non-coding RNAs on lung molecular clock. This review focuses on the ways how these non-coding RNAs regulate and in turn are regulated by the lung molecular clock and its potential impact on lung diseases.

## 1. Introduction

The physiology and behavior of mammals are subject to daily oscillations driven by an endogenous circadian clock that consists of a central pacemaker in the brain’s suprachiasmatic nucleus (SCN). The central pacemaker synchronizes subsidiary clocks in nearly every peripheral tissue tested [1]. Circadian rhythms (circa = about, dies = day) are near 24-h autonomous, self-sustained, biologic oscillations mediated by changes in the expression of clock genes [2]. Circadian rhythms are entrained to environmental cues, such as the day-night transition [2,3]. While the light-dark cycles are the predominant Zeitgebers (timing cues) for the central pacemaker, the peripheral pacemaker is often regulated and easily disrupted by metabolic status or environmental stimuli [4]. At the cellular level, circadian rhythmicity involves the molecular clock: a group of clock proteins that oscillate in a transcriptional-translational feedback loop. The central clock or central circadian pacemaker localized in the suprachiasmatic nucleus (SCN) of the hypothalamus in mammalian brain, was first reported by Mohawk et al., 2012 [2]. The central clock is entrained primarily by light followed by other physiological factors, such as feeding cues. Light enters the retina and signals via the retinal hypothalamic tract to the SCN. While most physiological processes were ‘driven’ by neural and hormonal output signals from the ‘central’ clock’, the discovery of ‘peripheral’ clocks within every tissue and organ of the body has provided a focus towards understanding the link between circadian disruption and diseases [5].

Non-coding RNAs (ncRNAs) play an important role in gene regulation. The most prominent non-coding RNAs are microRNAs that are involved in post-transcriptional gene silencing. Other prominent ncRNAs involved in gene health and disease include long ncRNAs and small nucleolar RNAs (snoRNAs). Dysregulation of these RNAs can have a pronounced impact on disease progression. NcRNAs play distinct roles in clock physiology. They are involved in regulation of core clock genes and clock output genes. Likewise, ncRNAs in turn are also regulated by clock genes in several organs. Core clock genes such as Brain and Muscle ARNT-Like 1 (BMAL1), CLOCK, Period (PER), Cryptochrome (CRY) and SIRT1 have all been shown to be regulated by miRNAs in several tissues [6]. To date none of the studies have looked at the potential effects of an aberrant microRNAome on the regulation of clock genes. Our results have shown that TGF-β1 a cytokine upregulated in smokers and COPD patients alters the bronchial epithelial microRNAome [7]. Some of these miRNAs are directly involved in regulating clock genes. It is well established that clock gene dysregulation in the lung promotes exacerbations in COPD as well as chronic inflammation [8,9,10]. This review discusses the reports of ncRNA mediated clock gene dysfunction observed in other tissues and its implications in lung diseases.

## 2. Mechanism of Circadian Clock

Both central and peripheral clocks use the same molecular machinery to ‘time’ the day. In each organ, interlocking repressing and activating transcriptional and translational feedback loops with a built-in ‘delay’ mechanism, culminate in the approximately 24-h rhythmic expression and activity of a set of core clock genes. In mammals, the molecular clock comprises at least ten genes that autoregulate each other in a transcriptional feedback loop [11]. The genes clock and BMAL1 (or Mop3) encode bHLH-PAS (basic helix-loop-helix; Per-ARNT-Single-mined) proteins that form positive limb of the feedback circuit [12]. CLOCK:BMAL1 heterodimers bind to the DNA promotor region of clock target genes at E-boxes (5′-CACGTG-3′) to initiate transcription [13,14,15]. A set of genes transcribed by the CLOCK/BMAL1 are the PER (PER1 & PER2) and CRY (CRY1 & CRY2) genes. PER and CRY genes form the negative arm of the autoregulatory feedback loop [13,16,17]. Heterodimers of PER and CRY dimerize in the cytoplasm, followed by nuclear translocation where they inhibit CLOCK:BMAL1 transcriptional activity allowing the cycle to repeat [18,19,20,21].

The CLOCK protein with intrinsic histone acetyl transferase (HAT) activity associates with BMAL1 and binds to E-box elements to drive the expression of clock output genes, REV-ERBα/β and negative regulators PER and CRY genes, which in turn suppress the expression of BMAL1. CLOCK acetylates the H3 and H4 making the chromatin epigenetically favorable for transcription of downstream genes. CLOCK also acetylates BMAL1 and PER2 (recruited by Ac-BMAL1). Ac-PER2 recruits Sirtuin1 (SIRT1) which deacetylates histones, PER2 and BMAL1. This dissociates PER2 reverting to a repressive chromatin state. PER2 dissociation leads to dissociation of SIRT1 and the cycle is repeated [22]. The rhythmic deacetylation of histones at the circadian gene promoters by SIRT1 is sensitive to NAD^+^ levels [23,24]. Rutter et al. [25] have shown that the NAD^+^:NADH ratio plays an important role in binding of CLOCK:BMAL1 to DNA.

The PER/CRY heterodimer also serves as an activator complex and also drives the expression on the nuclear receptors REV-ERBα/γ and RORα/γ (Retinoid-Related Orphan Receptor) [26]. These two receptors stabilize the oscillator by regulating the timing and amplitude of BMAL1 expression. Clock proteins are profoundly influenced by posttranslational modifications that affect both their activity and stability [23,27]. Outputs from the molecular clock are generated through transcription or repression of target genes. BMAL1 is regulated by rhythmic coordination with REV-ERBα, a nuclear hormone receptor and clock output gene. REV-ERBα is a critical regulator of inflammation and metabolism. REV-ERBα function is modulated by small-molecule ligands and thus represents an exciting option for manipulation of the clock in disease states [28,29]. Although clock-controlled genes (CCGs) are called primary clock proteins, the molecular oscillator also dictates the timing of tissue and cell specific genes [30]. These tissue and cell specific clock gene regulated genetic programs are the hands of the clock, facilitating its temporal program on systems physiology. Therefore, disruption of clock gene expression inevitably disturbs the timing and amplitude of these CCGs and the physiological processes which they control and has been implicated in chronic diseases [31,32]. The lung function demonstrates a robust diurnal rhythm, with a daytime peak (12:00 h) and early morning minimum (04:00 h) in healthy individuals [4]. Moreover, the early morning decline in lung function concurred with increased risk of exacerbations among patients with COPD and asthma [1,4]. Furthermore, lung molecular clock plays a significant role in the pathophysiology of chronic lung disease [33].

### 2.1. Circadian Molecular Clock in Lung Related Diseases

Circadian regulation of respiratory function has been described in both, experimental animals [34,35] and healthy humans [36,37]. Sukumaran et al. [38] reported over 600 genes showing robust oscillations in gene expression with over two thirds of them expressed during the dark cycle, including genes of lung homeostasis and repair. Mice conditioned to chronic jet lag or shift work conditions led to disruption of clock gene expression in the lung. Moreover, expression of clock genes demonstrated sexual dimorphism with male and female mice resulting in different levels of clock and BMAL1 expression under these conditions [35].

Obstructive lung diseases like Asthma, COPD demonstrate time-of-day variations in respiratory symptoms, including nocturnal breathlessness, insomnia and an early morning decline in lung function exacerbated with increased cough and mucus hypersecretion [39,40,41]. The pathological hallmarks of obstructive lung diseases like mucus hypersecretion, bronchodilator responses, surfactant protein levels, steroid efficacy and chronic cough likewise demonstrate circadian patterns [8,29,35,37,42,43,44,45].

The club cells in the bronchial epithelium are primarily involved in maintaining the pulmonary immune response in the lung [29]. CXCL5, a neutrophilic chemokine is expressed by club-cells and its expression is clock-controlled and demonstrates time-of-day variations. Gibbs et al. [29] showed that this is due to binding of the glucocorticoid receptor (GR) to its binding sites on the CXCL5 promoter. Expression of GR is clock-controlled and binding of GR to CXCL5 suppresses its expression. These observations by Gibbs et. al. [29] possibly explain the increased frequency of exacerbations observed in COPD and Asthma. Ablation of BMAL1 leads to enhanced CXCL5 expression even in presence of normal levels of GR, resulting in exaggerated inflammatory responses to lipopolysaccharide and bacterial infection [29]. Deletion of BMAL1 or mimicking jet lag conditions in mice, led to increased susceptibility to viral replication, increased exacerbation of acute viral bronchiolitis, mucus hypersecretion and increased airway resistance [46]. Likewise, suppression of REV-ERBα has been associated with increase in pro-inflammatory cytokines and augmented inflammatory responses [47].

**Figure 1:**
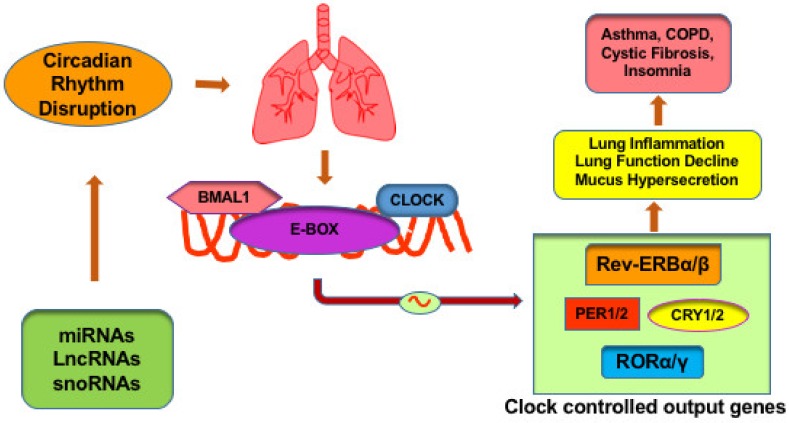
Schematic representation of ncRNAs regulation of lung molecular clock. Clock-controlled output genes are involved in lung function homeostasis. Outputs from the molecular clock are generated through transcription or repression of target genes. Clock genes plays a significant role in the lung pathophysiology of inflammation and metabolism. In the lung, disruption of these genes has been shown to promote exacerbations in chronic obstructive pulmonary disease (COPD), asthma, mucus hypersecretion, nocturnal breathlessness, insomnia as well as chronic inflammation.

As discussed above, Sirtuin 1 (SIRT1), an NAD^+^ -dependent deacetylase, affects the clock function by binding with CLOCK:BMAL1 complexes and subsequent deacetylation of BMAL1 and PER2 proteins [23,24,48,49,50]. SIRT1 activity is reduced in diseased states like COPD or upon exposure to cigarette smoke [51,52,53]. Acquired SIRT1 suppression upon exposure to cigarette smoke leads to increased acetylation of circadian clock proteins, culminating in abnormal clock gene expression and proinflammatory response in the lung [9]. Hence SIRT1 plays an important role in the lung molecular clock and its disruption has been linked to inflammation in lung diseases. Figure 1 shows a schematic representation of the effects of molecular clock dysregulation in lung diseases.

### 2.2. Role of Non-Coding RNAs in Molecular Clock Regulation: Implications to the Lung

**Figure 2:**
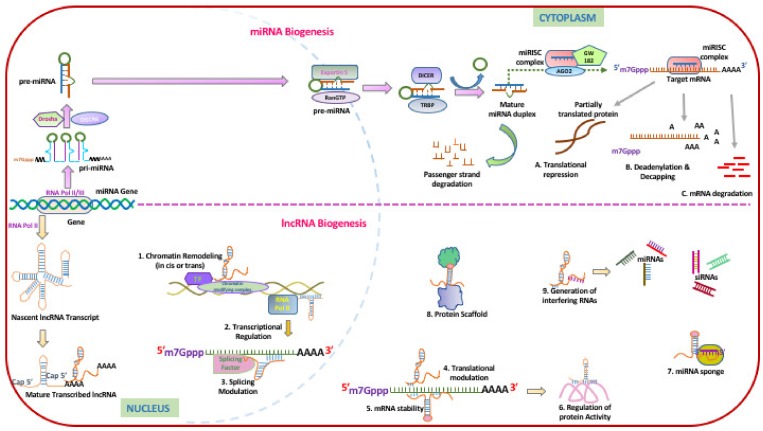
miRNAs and lncRNAs biogenesis and their role in epigenetic, transcriptional and posttranscriptional regulation. MiRNAs are short, single-stranded endogenous non-coding RNAs, of about 22 nucleotides that post-transcriptionally regulate gene expression. Most of the miRNA coding genes are found in introns and some are located as independent single transcriptional units or in clusters. miRNAs are involved in nearly all normal and developmental and pathological processes in humans. The miRNA biogenesis starts with transcription of gene into large primary transcript called pri-miRNA, which have 5′ caps and 3′ poly(A) tails2 and is typically mediated by RNA polymerase II and also some pre-miRNAs are generated by RNA polymerase III. Primary miRNA transcripts are processed into precursor miRNA (pre-miRNA) stem-loops of 60 nucleotides in length by the nuclear RNase III enzyme Drosha and its partner DGCR8. The transport of the pre-miRNA is mediated by the RanGTP-dependent nuclear transport receptor exportin-5 (EXP5). In cytoplasm, pre-miRNA are further cleaved by the endoribonuclease Dicer to mature ∼22 nt long miRNA–miRNA *duplex. After the duplex is unwound, the guide strand, is then loaded with Argonaute (Ago2) proteins into the miRNA-induced silencing complex (miRISC), where it binds to target mRNA by partial complementarity with its 3’UTR. This results in translational inhibition, mRNA degradation or deadenylation/decapping of the recognized mRNA target. lncRNAs are heterogenous regulatory elements comprise >200 nucleotides in length, and poorly conserved transcribed by RNA polymerase II, capped at the 5′ end, and polyadenylated at the 3′end. lncRNAs can be classified as intergenic, intronic, exonic, antisense and overlapping based on their genomic location. lncRNAs have significant role in many biologic processes such as cellular development, differentiation and survival. Changes in lncRNAs expression has implicated in the development of disease. 1. lncRNAs act as guide by recruiting chromatin-modifying enzymes to target genes, either in cis or in trans to distant target genes. 2. lncRNAs modulate the gene expression either by interacting with transcriptional activator or interacting with transcriptional repressor thereby promoting and suppressing their transcription, respectively. 3. lncRNAs regulate the mRNA alternative splicing by associating with slicing factors. 4. lncRNAs influence the protein translation through interactions with binding of translation cofactors and regulators. 5. lncRNA:mRNA interaction provides mRNA stability. 6. lncRNAs influence post-translational protein modification, alter protein localization, regulate protein activity or act as components of protein complex. 7. lncRNAs can act as miRNA sponges, influence the expression levels of the endogenous miRNA targets. 8. lncRNAs serve as protein scaffolds, forming ribonucleoproteins and bringing proteins in proximity to organize nuclear architecture. 9. In addition, few lncRNAs act as miRNA or small interfering RNAs (siRNA) precursor.

Non-coding RNAs can be considered important mediators of lung inflammation by virtue of their ability to regulate the expression of core clock genes as well as clock output genes thereby making them important mediators in inflammation and diseases. While microRNAs constitute the most studied non-coding RNAs and have been known to regulate clock gene expression by post-transcriptional gene silencing, there is increasing evidence for the role of other non-coding RNAs like long non-coding RNAs, and snoRNAs involved in maintaining or disrupting the molecular clock. Figure 2 shows the biogenesis of miRNAs and Long ncRNAs.

### 2.3. Role of MicroRNAs in Molecular Clock Regulation: Implications for the Lung

MicroRNAs (miRNAs) are a class of short (20–23-nucleotide), endogenous, single-stranded non-coding RNAs that play important roles in regulating the expression of target genes by directly binding to their mRNAs [54,55]. miRNA biogenesis is a sequential action of both nuclear and cytoplasmic processes. They are first transcribed as long primary miRNAs (pri-miRNAs), which are processed into precursor miRNAs (pre-miRNAs) by the enzyme drosha in the nucleus, followed by transport to the cytoplasm by RNA export factor Exportin 5 (XPO5) [56]. In the cytoplasm, the pre-miRNAs are further processed by DICER to ~20–22 bp mature miRNA duplexes with 2-nt 3′ overhangs [57,58,59,60,61]. The miRNA duplex is then incorporated into an Argonaute (Ago) proteins (AGO1, AGO2, AGO3 or AGO4) to form a miRNA-induced silencing complex (miRISC) [62,63] which subsequently mediates post-transcriptional gene silencing of the cognate RNA. miRNAs are also involved in transcriptional gene silencing whereby miRNAs are loaded to a specialized RNA-induced transcriptional silencing (RITS) complex, which consists of Ago1, and downregulates mRNA expression by chromatin remodeling [64].

miRNAs play an important role in regulating molecular clock. miRNAs can regulate clock genes or be regulated by clock genes, providing circadian regulation of their cognate targets. Shende et al. reported that microRNAs mir-142-3p and miR-494 directly target Bmal1 and regulates the circadian expression in the suprachiasmatic nuclei (SCN) [65,66]. The miR- 142-5p and miR-142-3p miRNAs play an important role in negative feedback regulation of molecular clock, with miR-142-3p and miR-142-5p suppressing BMAL1 and SIRT1, respectively [67,68,69]. MiR-142 expression demonstrates circadian rhythmicity and is CLOCK-controlled, with CLOCK:BMAL1 binding to upstream E-box elements of miR-142 [55,69]. Hence miR-142 serves as a core clock-controlled miRNA.

miR-17-5p has been shown to directly regulate the CLOCK gene. The regulation is reciprocal in that CLOCK protein directly binds to the promoter region of miR-17 and activates expression of miR-17-5p which in turn binds to the 3′UTR of CLOCK and suppresses its expression [70]. A number of microRNAs have been reported to target the PER genes of the molecular clock. Nagel et al. uncovered a microRNA cluster named miRNA-192/194 cluster that directly modulates the central components of the circadian clock [71,72]. Using a target-based screening method, they identified that endogenously expressed miR-192/194 cluster functions as a potential regulator and inhibitor of the entire Period gene family (PER1, PER2 and PER3). 3′-UTR of all PER genes contain putative target sites for miR-192/miR-194. The miR-34a-5p targets CRY1, PER1 and PER2) of molecular clockwork [59,73,74,75]. Likewise, miR-24 and miR-29b have been shown to directly suppress PER2 and PER3, respectively [59,76]. miR-29a and 30a are involved in regulating PER1 and PER2 [77]. Inhibition of PER genes has been shown to shorten the length of the circadian period and leads to an altered circadian cycle [72,78,79]. It is possible that this can lead to an increase in REV-ERBα levels leading to suppression of the tumor suppressor miR-122, since REV-ERBα is important in the post-transcriptional tuning of miR-122 [80,81]. Although the mature form of miR-122 expression does not oscillate, the expression of both pri-miR-122 and pre-miR-122 displays circadian oscillations [81]. miR-122 is an important tumor suppressor miRNA, as it inhibits tumor cell proliferation, induces apoptosis and suppresses metastasis in non-small cell lung cancer (NSCLSC) [60,61]. Shilts et. al. have shown that REV-ERBα expression is altered in lung cancer [82].

Na et al. [83] demonstrated that miR-181d and miR-191 exhibited inversely correlated circadian rhythm by controlling the circadian initiators, clock and Bmal1 in mouse liver [83]. In colorectal cancer, miR-181d plays an oncogenic role by directly targeting the 3′-UTRs of CRY2 and FBXL3 and by promoting glycolysis which implies the notion that dysregulated expression of clock gene leads the formation of tumor [84,85]. Cancer progression is also influenced by the circadian system whose functioning is based on the rhythmic expression of clock genes.

### 2.4. TGF-β in Altered microRNAome and Its Impact on Clock Regulation: Implications to the Lung

TGF-β1 expression is upregulated in the airways of smokers as well as COPD patients [86,87,88,89]. TGF-β signaling has been shown to disrupt clock gene expression in many cell types. For instance, TGF-β has been shown to suppress the expression of PER1, PER2 and REV-ERBα [90]. TGF-β1 itself has been shown to upregulate BMAL1 in lung epithelial cells as well as fibroblasts [91]. TGF-β itself is subject to circadian control by BMAL1 and CLOCK genes [91,92]. We have demonstrated that TGF-β1 signaling upregulates multiple miRNAs capable of disrupting the lung molecular clock. We have observed that TGF-β induces four miRNAs capable of targeting SIRT1, namely miR-449a [93,94] (2-fold or Log2 of 1.0), miR449b-5p [93] (7.07-fold or Log2 of 2.8), miR-126–3p [95] (2.5-fold or Log2 of 1.32) and miR-34a-5p [96,97] (3.7-fold or Log2 of 1.91). While only one of the miRNA, miR-449b made the cut off of Log2 of 2 (4-fold) in our manuscript [7], more than one miRNAs, upregulated at lower thresholds can have a profound impact on the specific target gene expression. Indeed, we have observed that TGF-β1 suppresses SIRT1 expression in primary human bronchial epithelial cells (unpublished data). The aberrant microRNAome induced by TGF-β1 in bronchial epithelial cells can directly impact core clock genes as well as clock output genes. We have demonstrated that TGF-β1 treatment leads to a 1.78-fold (Log2 of 0.84) decrease in miR-140–5p known to target REV-ERBα [7]. It is possible that this can lead to an increase in REV-ERBα levels leading to suppression of the tumor suppressor miR-122.

Another clock controlling miRNA upregulated in our studies is miR-29b-3p (3.6-fold or Log 2 of 1.85) that targets PER1 [98]. TGF-β1 upregulates miR-33b-5p (24.77-fold or Log2 of 4.63 [7]) known to suppress the clock output gene RORα [99]. TGF-β1 also suppresses another miRNA known to regulate RORα, namely miR-137 (7.6-fold or Log2 of 2.92 [7,100]). While suppression of miR-137 would be expected to increase the levels of RORα, it is possible that the net result of these counteracting miRNAs is RORα suppression given the increased magnitude of miR-33b-5p upregulation. We have already shown that cigarette smoke and HIV Tat induce TGF-β1 signaling [7,101,102]. Hence it is possible that the bronchial epithelial microRNAome is disrupted in smokers and people living with HIV, leading to a dysfunctional lung molecular clock. This can manifest as chronic inflammation and increased exacerbations in obstructive lung diseases.

### 2.5. Role of Non-Coding RNAs in Molecular Clock Regulation: Implication for the Lung

Long non-coding RNAs (lncRNA) are mRNA like structures which lack protein coding potential. They can be located both in nucleus and cytoplasm, with a size of over 200 nucleotides. LncRNAs have been implicated in regulating various cellular functions by participating in chromatin modifications, mRNA decay, alternative splicing and transcription through cis and trans mechanisms [103] and are implicated in chronic lung diseases. In recent years, lncRNAs have also been shown to regulate various molecular pathways of the cellular circadian clock via an intriguing circadian-chromatin regulatory network. A pioneering study by Coon et al. [104] reported the association of lncRNAs with mammalian circadian rhythms while investigating the molecular regulation of melatonin hormone that helps to control the biologic rhythms of sleep-wake cycle. The RNA-Seq analysis of the rat pineal glands (an endocrine gland that produces and secretes melatonin), revealed 112 differentially expressed lncRNAs of which eight lncRNAs including lncSN001, lncSN004, lncSN012, lncSN016, lncSN056, lncSN081, lncSN134 and lncSN215 showed differential night/day expression.

Analysis of mammalian liver epigenome also revealed a significant association of a number of lncRNA species with circadian oscillations of metabolism regulating genes. Widespread changes in histone modifications but not DNA methylation showed strong association with transcriptional oscillations which included 1262 murine hepatic oscillating transcripts. These included 123 lncRNAs that were differentially expressed, as analyzed by deep RNA sequencing [105]. Among 123 lncRNAs, 19 lncRNAs showed robust transcriptional oscillations, indicating a synergistic role of lncRNAs with the histone modifications in regulating the circadian nature of gene expression.

Circadian oscillations regulate metabolism and physiology by multiple mechanisms. These diurnal rhythmic perceptions by the peripheral tissues such as retinal photoreceptor cells are critical in anticipating the required metabolic shifts. To understand the circadian process of the photoreceptor disc shedding and the retinal pigment epithelial (RPE) cell-mediated phagocytosis, RNA-Seq analysis of mouse retina was conducted [106]. Besides the core clock genes 191 differentially expressed lncRNAs were identified of which 16 showed strong association with the oscillating expression pattern. A detailed analysis of lncRNA ENMUST00000138486 revealed its location near the Mertk loci along with a 5-kb promoter region, containing the promoter motifs for binding of CLOCK:BMAL1 and REV-ERBα [106].

As the circadian clock governs cell physiology and cell metabolism, any disruption in these diurnal rhythms results in accelerated aging and aging-associated comorbidities. Several lncRNA were shown to regulate aging and associated circadian rhythms [107]. The RNA-Seq of 4, 12 and 20 months old of zebrafish brain tissues analyzed at 2 diurnal points and the clustered analyses revealed differentially expressed 17,702 transcripts, where 6 lncRNAs showed noticeable diurnal oscillation at 12 months of age. Similar expression pattern was also noted with histone H3 lysine 9 tri-methylation (H3K9me3) that coincided with age- and diurnal-related changes. Subsequently, in another study using the zebrafish brain and the mice liver, a lncRNA Telomeric Repeat-containing RNA (TERRA) was found to be associated with circadian transcription factor BMAL1. Ablation of BMAL1 expression in a mouse model resulted in the disruption of TERRA’s diurnal rhythmic expression and H3K9me3-mediated telomeric homeostasis, thus implicating, TERRA and BMAL-1 in aging-related telomeric erosion [108].

Similarly, the circadian clock disruption and the metabolic shift, both regulated by various lncRNAs, has also been associated with cancerous transformations. The impact of clock associated lncRNAs in hepatocellular carcinoma [109] showed that the lncRNA HULC upregulates the expression levels of CLOCK and its downstream targets, like PER1 and CRY1 in hepatoma cells in vivo. Bioinformatic analysis and the experimental validation performed in glioma, a lethal malignant brain tumor, showed that lncRNA UCA1 regulate CLOCK genes via miR-206 [110]. This UCA1/miR-206/CLOCK axis was implicated to show the integrative effect on proliferation and cell cycle of glioma cells.

Analysis of a metabolic disorder, non-alcoholic fatty liver disease (NAFLD showed that in addition to the significant amounts of mRNA transcripts, 291 lncRNA species were differentially expressed in the diseased conditions [111]. The GO and KEGG pathway analysis revealed 8 lncRNAs named fatty liver-related lncRNAs, FLRLs (FLRL1-8), to be associated with the circadian rhythm regulation. Further qPCR validation analysis confirmed that the lncRNAs FLRL1, FLRL2 and FLRL6 were involved in circadian clock by targeting the PER3, BMAL1 and PER2 genes, respectively.

Molecular clock and the lncRNAs has also been shown to regulate the cardiovascular physiology and endothelial system. The lncRNA NEAT1 inhibits the proliferation of human umbilical vein endothelial cells (HUVECs) when exposed to Trimethylamine N-oxide (TMAO), a chemical inducer of atherosclerotic development. TAMO induced the lncRNA NEAT1 which further regulates CLOCK:BMAL1 expression. Knocking down NEAT1 expression downregulates the CLOCK expression whereas the NEAT1 overexpression leads to upregulation of BMAL1 [112]. The regulatory role of lncRNA NEAT1 on CLOCK:BMAL1 gene expression was also confirmed using a NEAT1 targeted genetic editing approach in vascular smooth muscle cells. The cells were treated with acrolein, to induce cell death to model atherosclerotic plaque development, also considered as a circadian disorder [113].

### 2.6. Role of Small Nucleolar RNAs (snoRNAs) in Molecular Clock Regulation: Implications to the Lung

SnoRNAs are 60–300 nucleotide long non-coding RNAs primarily involved in the modification and processing of ribosomal RNA. These RNAs are primarily grouped into the C/D box snoRNAs which methylates the 2′-hydroxyl groups of the pre-rRNA precursor, and the H/ACA box snoRNAs which are involved in the modification of uridines to pseudouridines. snoRNAs act as scaffold to assemble protein complexes and guide the ribonucleoprotein complex to the target RNA mainly rRNA, using base-pairing between the snoRNA and its target [114,115,116,117]. Aitken and Semple showed that 18S and 28S pre-rRNA demonstrate circadian regulation that negatively corelates with expression of snoRNAs involved in modifying the rRNA precursor [118]. Another seminal study on the Prader-Willi syndrome (PWS), a genetic disorder of obesity, intellectual disability and sleep abnormalities [119], identified that PWS locus encompasses SNORD116 small nucleolar RNA (snoRNA) and a spliced lncRNA 116HG, which acts as a key regulator of diurnal energy expenditure of the brain. They followed fluorescent in-situ RNA/DNA hybridization (FISH) and chromatin isolation by RNA purification (ChiRP) approaches to show that the subnuclear lncRNA 116HG binds to RBBP5 (retinoblastoma binding protein 5) and stimulates histone methyl transferase activities. Deletion of SNORD116 in a mouse model lacking the lncRNA 116HG, exhibited increased energy expenditure due to dysregulation of the diurnally expressed mTor and the clock, CRY1 and PER2 circadian genes [119,120]. SNORA42 has been implicated in lung cancer [121] and many snoRNAs serve as biomarkers for lung cancer [122]. However, their effect on the lung molecular clock or their circadian regulation remains undiscovered.

## 3. Conclusions

Peripheral clocks play an important role in maintaining organ homeostasis; disruption of the peripheral clock can lead to disease states. While a number of studies have demonstrated the role of ncRNAs like miRNAs and long ncRNAs in regulating core clock genes as well as clock output genes in other organs, surprisingly none of the studies have explored a similar ncRNA-mediated disruption of the molecular clock in the lung. The aberrant microRNAome induced by TGF-β signaling on the bronchial epithelium includes multiple microRNAs that can disrupt the lung molecular clock by suppressing or activating core clock genes as well as clock output genes. The effects of genes on inflammation and lung disease-like maladies has been extensively reviewed elsewhere [33]. This review provides a first peek at the possible pathophysiological mechanisms relating an aberrant microRNAome or dysregulated expression of long ncRNAs to lung circadian disruption with consequent inflammation and lung diseases.
